# Insulin Resistance Indices and Subclinical Left Ventricular Systolic Dysfunction in Adults: A Systematic Review and Meta-Analysis

**DOI:** 10.31083/RCM49364

**Published:** 2026-07-24

**Authors:** Hao Li, Yuke Ma, Lei Zhao

**Affiliations:** ^1^Department of Cardiology, Affiliated Hospital of Jining Medical University, 272000 Jining, Shandong, China; ^2^Department of Neurology, The Second Hospital of Jilin University, 130000 Changchun, Jilin, China; ^3^Department of Cardiology, The Second Hospital of Jilin University, 130000 Changchun, Jilin, China

**Keywords:** insulin resistance, triglyceride-glucose index, global longitudinal strain, speckle-tracking echocardiography, subclinical ventricular dysfunction

## Abstract

**Background:**

Insulin resistance (IR) is a key characteristic of cardiometabolic disease and may contribute to subclinical ventricular dysfunction before the development of overt heart failure. Studies have reported heterogeneous results regarding the associations between IR indices and myocardial strain. Thus, this review synthesized the available evidence on the relationships between IR indices and subclinical left ventricular systolic dysfunction in adults.

**Methods:**

We systematically searched PubMed, Embase, Web of Science Core Collection, and the Cochrane Library from inception to 1 December 2025 for observational studies involving adults that reported associations between IR indices and speckle-tracking echocardiography-derived strain parameters. Eligible exposures included commonly used IR indices, such as the homeostatic model assessment of insulin resistance (HOMA-IR), the triglyceride-glucose (TyG) index, the metabolic score for insulin resistance (METS-IR), and lipid-based ratios. The primary outcome was global longitudinal strain (|GLS|). Right ventricular strain was summarized narratively because only one study reported this condition. Adjusted odds ratios (ORs) for subclinical LV systolic dysfunction per unit increase in an IR index and mean differences (MDs) in |GLS| between the highest and lowest IR categories were synthesized using random effects models with Hartung-Knapp adjustment.

**Results:**

Four studies reporting fully adjusted ORs per unit increase in an IR index showed a consistent positive association with subclinical LV systolic dysfunction. The pooled OR per unit increase in the IR index was 1.36 (95% confidence interval (CI): 1.20 to 1.53; I^2^ = 0%). Five studies comparing GLS across IR quartiles reported that participants in the highest quartile had worse LV systolic function than those in the lowest quartile. The random-effects pooled MD in GLS (Q4 vs. Q1) was –1.58 percentage points (95% CI: –2.22 to –0.93), indicating a lower strain magnitude (worse subclinical systolic function) in the highest IR group.

**Conclusions:**

Higher IR indices were independently associated with lower GLS values, indicating worse subclinical LV systolic function. The association was directionally consistent across IR metrics, but the supportingevidence remains limited, prospective validation and interventional studies are needed.

**The PROSPERO Registration:**

CRD420251251563, https://www.crd.york.ac.uk/PROSPERO/view/CRD420251251563.

## 1. Introduction

Insulin resistance (IR) is a key pathophysiological feature of obesity, metabolic syndrome, and type 2 diabetes mellitus. It is considered an important determinant of cardiovascular risk in the general adult population [1,2,3]. Besides its role in the development of overt diabetes and atherosclerotic disease, IR is associated with myocardial remodeling, low-grade inflammation, and microvascular dysfunction, which together may help develop heart failure even before symptoms appear [2,4,5,6].

The assessment of IR in clinical and epidemiological studies relies mainly on surrogate indices derived from fasting glucose, insulin, and lipid parameters because the euglycemic-hyperinsulinemic clamp is too complex for routine use [7,8]. Commonly used indices include homeostatic model assessment of insulin resistance (HOMA-IR), the triglyceride-glucose (TyG) index, the triglyceride-to-HDL (high-density lipoprotein) cholesterol ratio (TG/HDL), and composite scores such as the metabolic score for IR (METS-IR) or the estimated glucose disposal rate [9,10,11,12]. These indices capture different aspects of glucose-lipid metabolism and are widely available in the general population and high-risk populations, including but not limited to individuals with type 2 diabetes.

Subclinical ventricular dysfunction can now be detected by speckle-tracking echocardiography before changes in the left ventricular ejection fraction become evident [13]. The global longitudinal strain (GLS) of the left ventricle is more sensitive than conventional parameters for identifying early systolic impairment, and right ventricular free-wall longitudinal strain provides complementary information on right ventricular performance [14,15]. Several single-center studies have found that higher levels of IR indices are associated with less negative left ventricular global longitudinal strain (LV GLS) and impaired right ventricular strain in adults from various clinical settings, including community cohorts, obesity or metabolic syndrome, and diabetes clinics. However, these studies have differences in design, sample size, IR indices used, and whether they evaluate the left ventricle, and many are underpowered to quantify dose-response relationships across IR categories [16,17,18,19,20,21,22].

The extent to which different IR indices are consistently associated with subclinical left ventricular systolic dysfunction and whether these associations are similar in the general population and in specific high-risk subgroups, such as patients with type 2 diabetes, remain unclear. Therefore, we conducted a systematic review and meta-analysis of observational studies involving adults to synthesize evidence on the relationships between IR indices and speckle-tracking-derived measures of subclinical left ventricular systolic dysfunction, with additional analyses in key clinical subgroups, including individuals with and without type 2 diabetes.

## 2. Methods

We developed the protocol a priori and registered it in PROSPERO (CRD420251251563, https://www.crd.york.ac.uk/PROSPERO/view/CRD420251251563). This review was reported in accordance with PRISMA [23]. We searched PubMed, EMBASE, Web of Science, and the Cochrane Library from inception to 1 December 2025 without language restrictions. The search strategy combined controlled vocabulary (for example, MeSH) and free-text terms and was structured around two concept blocks: IR (and IR indices) and myocardial strain assessed by strain echocardiography. The terms for IR included HOMA-IR, TyG, METS-IR, the metabolic score for IR, TG/HDL, and the estimated glucose disposal rate; strain-related terms included global longitudinal strain, left ventricular strain, right ventricular free-wall longitudinal strain, myocardial strain, speckle-tracking echocardiography, and strain echocardiography. Two reviewers (H.L. and Y.M.) independently conducted the searches and screened records; any discrepancy was resolved by consulting a third reviewer (L.Z.). The reference lists of eligible original studies and relevant review articles were hand-searched. The full search strategy for each database is provided in** Additional file 1**

### 2.1 Inclusion and Exclusion Criteria

Studies were considered to be eligible if they met all of the following criteria: (1) evaluated the association between an IR index and subclinical left ventricular systolic dysfunction; (2) defined subclinical ventricular dysfunction using imaging-derived measures of myocardial function; and (3) reported an effect estimate (odds ratio (OR)/RR/HR with 95% confidence interval (CI)) for subclinical LV dysfunction or reported strain values (mean ± standard deviation (SD)/SE or data convertible to mean ± SD) across IR categories sufficient to calculate mean differences (MDs). Studies were excluded if they (1) included only patients with established, clinically overt heart failure at baseline; (2) were conducted with non-human subjects; (3) did not provide extractable data for pooling; or (4) were not original research articles.

### 2.2 Data Extraction and Quality Assessment

Two reviewers (H.L. and Y.M.) independently extracted the data using a standardized form. Any discrepancy was resolved by discussion with a third reviewer (L.Z.). For each study, the following information was collected: first author, year of publication, study design, study setting/country, sample size, participant characteristics, the IR index evaluated (including how it was modeled), covariates included in multivariate analyses, and the reported effect estimate. The quality of the study was appraised using a modified Newcastle–Ottawa Scale (NOS: range 0–9 points) [24]. Studies with NOS scores ≥6 were considered to be of higher methodological quality. The modified NOS items and scoring criteria are summarized in **Supplementary Table 1**.

### 2.3 Statistical Analysis

GLS is frequently reported as a negative percentage, where more negative values indicate better systolic function. To ensure a consistent direction of effect across studies, we harmonized all strain measures to the absolute GLS magnitude (|GLS|), expressed as positive percentages. Therefore, a higher |GLS| indicates better systolic function. Group differences were defined such that a negative MD reflects a lower |GLS| (worse systolic function) in the higher IR group. For each comparison, MD was calculated as follows: MD = |GLS| in the higher-IR group minus |GLS| in the lower-IR group.

When studies reported GLS or right ventricular free-wall longitudinal strain as the mean ± SD, these values were directly used. When the data were presented as medians (with interquartile ranges) or medians (with ranges), we approximated the means and SDs using established methods [25]. If strain was provided separately for multiple categories of an IR index (quartiles), we primarily extracted the contrast between the lowest and highest categories (Q1 vs. Q4) to capture the maximal gradient in ventricular function while avoiding multiple counting of the same participants. For studies reporting more than one IR index or both left and right ventricular strain, each index-outcome pair was included in the corresponding meta-analysis, and pre–specified sensitivity analyses were performed to address potential unit-of-analysis issues.

For each study and comparison, the MD and its standard error were computed using the inverse-variance method. Pooled effect estimates with 95% CI were obtained by conducting random-effects meta-analyses, with between-study variance estimated using the DerSimonian–Laird method. To obtain more robust uncertainty estimates, particularly for comparisons with a few studies (subgroup analyses), we applied the Hartung–Knapp–Sidik–Jonkman adjustment for CIs and hypothesis testing.

Between-study heterogeneity was quantified using Cochran’s Q test and the I^2^ statistic, which describes the percentage of total variability due to heterogeneity rather than chance. I^2^ values of about 25%, 50%, and 75% were considered to represent low, moderate, and high heterogeneity, respectively.

Several sensitivity analyses were conducted to assess the robustness of the findings: (1) we investigated potential sources by conducting prespecified subgroup analyses, stratified by (a) the type of IR index (TyG index and other IR index); (b) the definition of subclinical ventricular dysfunction (cutoff 18% and cutoff 20%); (c) the population type (diabetes mellitus and non-diabetes mellitus); and (2) publication bias and small-study effects, which were evaluated visually by funnel plots and statistically using Egger’s regression test. When there was evidence of asymmetry, the trim-and-fill procedure was used to estimate the potential effect of missing studies on the pooled effect. (3) Fixed-effect models were additionally fitted for sensitivity analyses. (4) Leave-one-out influence analyses were performed by sequentially omitting each study from the meta-analysis to examine the robustness of the pooled effect sizes.

All statistical analyses were conducted using R software, version 4.5.2 (R Foundation for Statistical Computing, Vienna, Austria). Meta-analyses were mainly performed using the meta and metafor packages. All tests were two-sided, and *p* < 0.05 was considered to be statistically significant.

## 3. Results

### 3.1 Literature Search and Selection

**Fig. 1.**
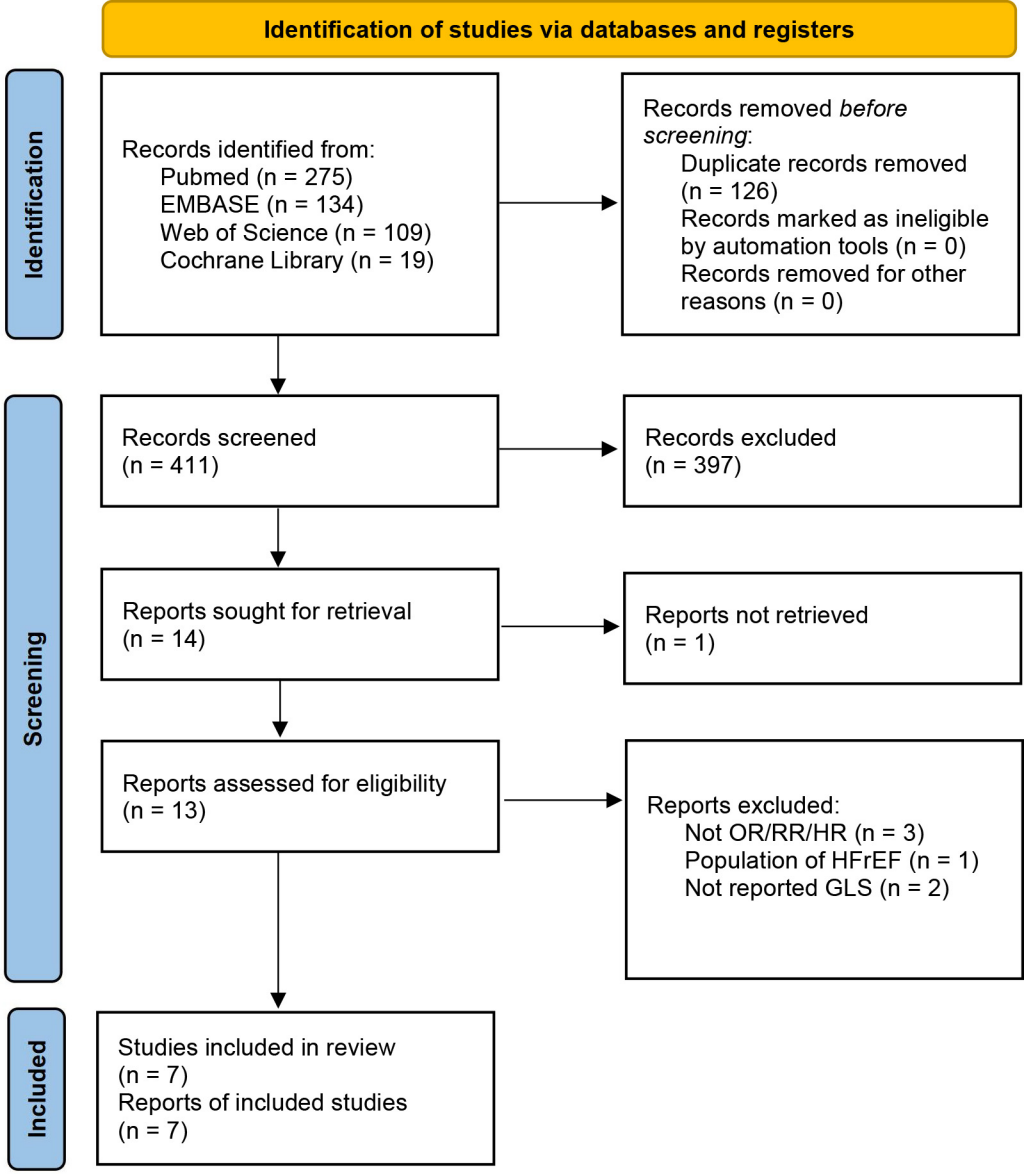
**Flow diagram of study selection**.

A total of 537 records were retrieved. After duplicates were removed, 411 records remained for title and abstract screening, of which 397 were excluded. 14 reports were sought for retrieval, and one was not retrieved. Therefore,13 full-text articles were assessed for eligibility. Among them, three did not report effect estimates (ORs/RRs/HRs) with sufficient information, one included only patients with heart failure with reduced ejection fraction (HFrEF), and two did not define subclinical ventricular dysfunction by imaging-based measures of myocardial function. Ultimately, seven studies were included in the systematic review; six studies reported the LV GLS and contributed to the quantitative synthesis, whereas the single RV strain study was summarized narratively (Fig. 1).

### 3.2 Study Characteristics and Quality Assessment

Seven observational studies published between 2023 and 2025 were included, comprising 4590 participants [16,17,18,19,20,21,22]. Six studies were conducted in China, and one was conducted in Italy. The study included patients with diabetes, obesity, and hypertension, and participants with preserved LVEF (>50%) in two studies. TyG was the most frequently investigated IR metric (five studies; n = 3958), followed by HOMA-IR (one study; n = 87) and the TG/HDL-C ratio (one study; n = 545). With respect to echocardiographic outcomes, six studies assessed LV GLS, whereas one study evaluated right ventricular free-wall longitudinal strain (RV FWLS) (Table 1, Table 2, Ref [16,17,18,19,20,21,22]). All studies reported multivariable-adjusted effect estimates, and the quality of the methodology was generally high (details of the NOS scoring can be found in **Supplementary Table 2**).

**Table 1.** **Characteristics of the included studies and adjusted effect estimates**.

Author	Design	Subclinical cutoff	IR analysis	Variables adjusted	Adjusted effect estimate (OR, 95% CI)
SUN et al. [16]	retrospective	<18%	Continuous	age, sex, BMI, systolic blood pressure and low-density lipoprotein cholesterol	TyG index was an independent risk factor for subclinical LV systolic dysfunction in type 2 diabetes mellitus patients (OR: 1.613, 95% CI: 1.004–2.590)
Li et al. [17]	cross-sectional	<20%	Continuous	age, sex, BMI, HR, hypertension, diabetes mellitus, dyslipidemia, blood urea nitrogen, serum creatinine, LV mass and LVH	TyG index remained an independent risk indicator related to an LV GLS <20% (OR: 1.520, 95% CI: 1.040–2.221)
Chen et al. [18]	cross-sectional	<18%	Categorized	age, gender, diabetes duration, systolic pressure, HbA1c, BMI, hypertension, heart rate, logarithmic microalbuminuria, LVEF and insulin therapy	The higher quartile of the TyG index remained an independent risk indicator related to GLS <18% (Q4 vs Q1: OR:5.23, 95% CI: 1.12–24.51)
Yang et al. [19]	cross-sectional	<18%	Continuous	gender, proteinuria, white blood cell count, percentage of neutrophils, blood urea nitrogen, eGFR, serum creatinine, uric acid, HDL, fasting C-peptide, HOMA, fasting blood glucose, triglycerides (combined as Tyg), RPP, age, obesity, hypertension, DM duration, smoking history	With the increase in IR, the probability of subclinical left ventricular systolic insufficiency in older adults with T2DM progressively increased (OR: 1.249, 95% CI: 1.027–1.521)
Zhou et al. [20]	cross-sectional	<18%	Categorized	age, sex, BMI, systolic blood pressure, heart rate, current smoking, alcohol intake, coronary artery disease, hypertension, diabetes mellitus, low-density lipoprotein cholesterol, estimated glomerular filtration rate, the use of antihypertensive, antihyperglycemic drugs, serum N-terminal pro-B-type natriuretic peptide, left ventricular mass index, and e′	The highest quartile of the TyG index had no significantly higher odds of subclinical LV systolic dysfunction compared to those in the lowest quartile after comprehensive multivariate adjustments (OR: 0.99, 95% CI: 0.63–1.57)
Cassano et al. [21]	cross-sectional	≤20%	Continuous	age, gender, Glycemic profile, Obesity, eGFR, hs-CRP, Matsuda index, LVMI, TAPSE/PAPS, E/e′	TG/HDL-C ratio is an independent risk factor for impaired myocardial deformation (OR: 1.381, 95% CI: 1.15–1.658)
Bian et al. [22]	retrospective	<20%	Continuous	age, sex, BMI, SBP, LV GLS, hypertension, and dyslipidemia	TyG index was related to RV FWLS <20% (OR: 1.888, 95% CI: 1.005–3.545)

IR, insulin resistance; OR, odds ratio; BMI, body mass index; TyG, triglyceride-glucose index; HR, heart rate; LV GLS, left ventricular global longitudinal strain; LVH, left ventricular hypertrophy; HbA1c, glycated hemoglobin; LVEF, left ventricular ejection fraction; eGFR, estimated glomerular filtration rate; HDL-C, high-density lipoprotein cholesterol; HOMA-IR, homeostatic model assessment of insulin resistance; RPP, rate-pressure product; DM, diabetes mellitus; LVMI, left ventricular mass index; TAPSE, tricuspid annular plane systolic excursion; PAPS, pulmonary artery systolic pressure; SBP, systolic blood pressure; RV FWLS, right ventricular free-wall longitudinal strain.

**Table 2.** **Characteristics of the participants**.

Author	Year	Study time	Region	Population	IR	High IR cutoff	Number of participants	Ventricle	Strain type
SUN et al. [16]	2023	2021.3– 2022.7	China	DM	TyG	≥9.49	183	LV	GLS
Li et al. [17]	2024	2019.1– 2024.1	China	Obesity	TyG	≥8.97	617	LV	GLS
Chen et al. [18]	2023	2021.6– 2021.12	China	DM	TyG	>9.83	150	LV	GLS
Yang et al. [19]	2025	2021.11– 2024.8	China	DM Age ≥60	HOMA-IR	NR	87	LV	GLS
Zhou et al. [20]	2025	2017.6– 2019.5	China	LVEF >50%	TyG	>9.0	2850	LV	GLS
Cassano et al. [21]	2025	NR	Italy	Hypertension	TG/HDL	>3.75	545	LV	GLS
Bian et al. [22]	2025	2020.8– 2024.6	China	DM LVEF >50%	TyG	>9.76	158	RV	FWLS

NR, not reported; DM, diabetes mellitus; FWLS, free-wall longitudinal strain; GLS, global longitudinal strain.

### 3.3 Ventricular Function According to IR Quartiles

**Fig. 2.**
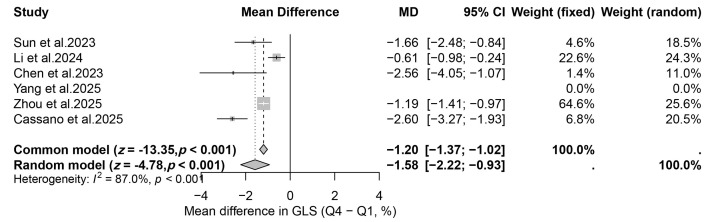
**Forest plot of the associations between IR indices and left ventricular global longitudinal strain (Q4 vs. Q1)**.

Five studies compared absolute LV GLS magnitude (|GLS|) between participants in the highest quartile (Q4) and the lowest quartile (Q1) of an IR index (Fig. 2). In all studies, participants in Q4 presented a lower |GLS| than those in Q1, with study-specific MDs ranging from –0.61% to –2.60% (MD = |GLS| in Q4 minus |GLS| in Q1). The pooled fixed-effect estimate indicated that |GLS| was, on average, 1.20 percentage points lower in Q4 than in Q1 (MD = –1.20%, 95% CI: –1.37 to –1.02; *p* < 0.001). Given the substantial between-study heterogeneity (I^2^ = 87.0%, *p* < 0.001), we prioritized the random effects model, which yielded a larger pooled difference (MD = –1.58%, 95% CI: –2.22 to –0.93; *p* < 0.001). These results suggested that adults in the highest IR category had lower |GLS|, which is consistent with worse subclinical LV systolic function, although the magnitude of impairment differed across studies.

### 3.4 Insulin Resistance Index and Subclinical Left Ventricular Systolic Dysfunction

**Fig. 3.**
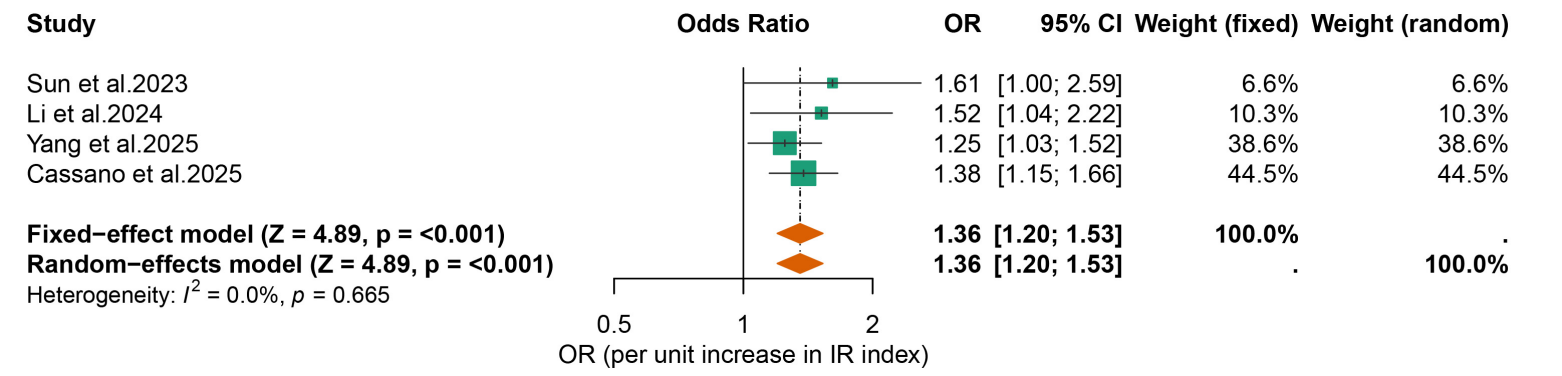
**Forest plot of the associations between IR indices and subclinical left ventricular systolic dysfunction**.

Four studies evaluated the fully adjusted odds ratio (OR) per unit increase in an IR index for subclinical LV systolic dysfunction (Fig. 3). Individual ORs ranged from 1.25 (95% CI: 1.03 to 1.52) to 1.61 (95% CI: 1.00 to 2.59). Using random-effects models with Hartung-Knapp adjustment, each unit increase in an IR index was associated with increased odds of subclinical LV systolic dysfunction (OR = 1.36, 95% CI: 1.20 to 1.53; I^2^ = 0%).

Two studies reported fully adjusted quartile-based ORs for subclinical LV systolic dysfunction and obtained inconsistent results. Given the limited number of studies and substantial between-study differences, these results are summarized descriptively in the Discussion section.

Prespecified subgroup analyses are reported in **Supplementary Materials** and should be interpreted as exploratory because of the small number of studies (**Supplementary Figs. 1–3**).

### 3.5 Publication Bias

**Fig. 4.**
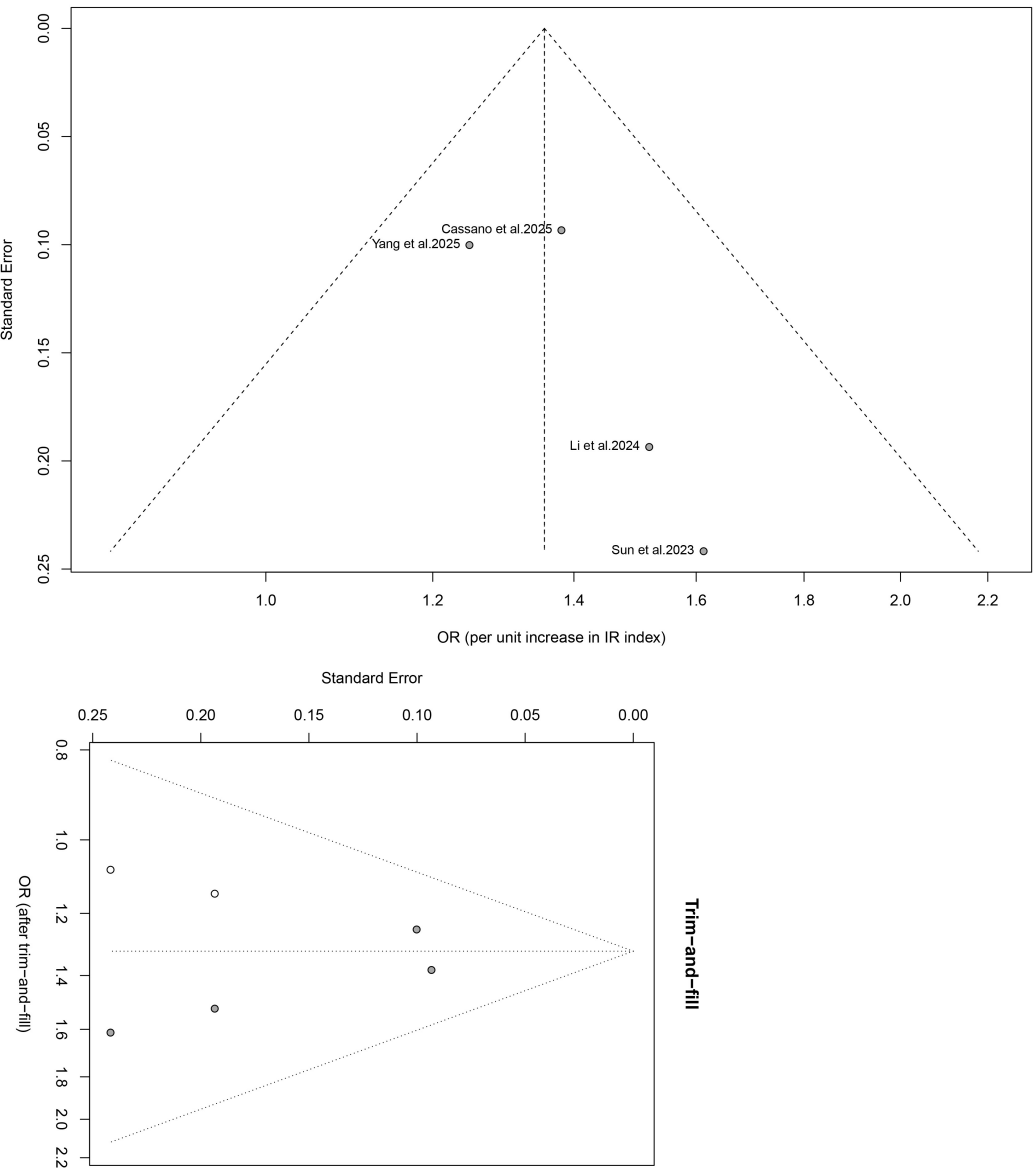
**Funnel plot for assessing publication bias (IR indices modeled as continuous variables)**.

Visual inspection of the funnel plot suggested no gross asymmetry (Fig. 4). Egger’s regression test for small-study effects was not significant (*p* = 0.232), although the number of included studies was small (k = 4), and the test was probably underpowered. The trim-and-fill procedure imputed two potentially missing studies. After adjustment, the pooled association remained similar to that in the main analysis (random effects model: OR = 1.32, 95% CI: 1.17–1.48). Given the small number of studies, trim-and-fill results should be interpreted cautiously and presented as exploratory.

### 3.6 Sensitivity Analysis

**Fig. 5.**
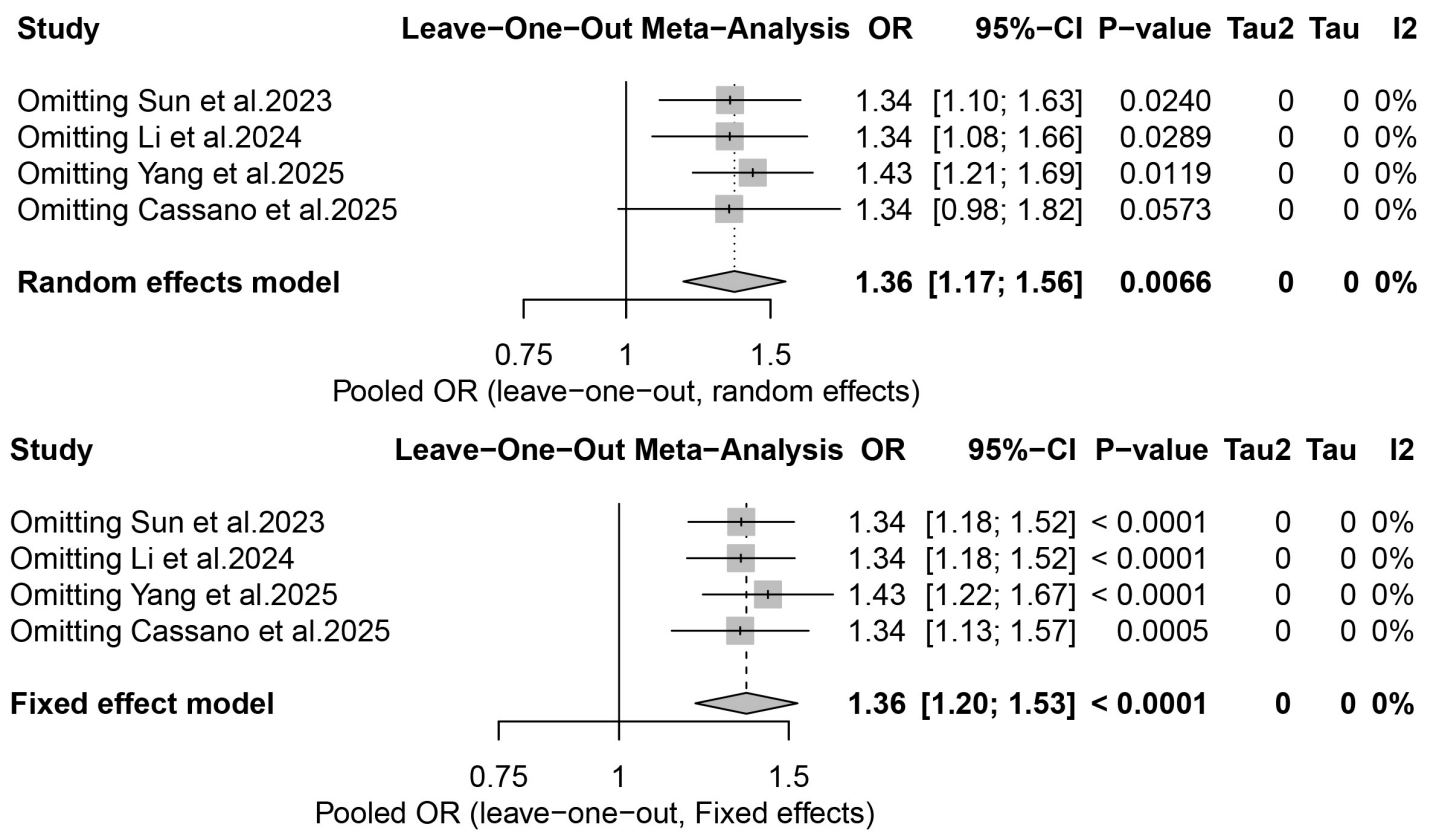
**Leave-one-out influence analysis for the associations between IR indices and subclinical left ventricular systolic dysfunction (continuous models)**.

In the leave-one-out influence analyses, sequential exclusion of each study did not materially change the pooled OR, suggesting that no single study unduly influenced the overall association (Fig. 5).

## 4. Discussion

### 4.1 Main Findings

In this systematic review and meta-analysis, higher IR was consistently associated with worse subclinical LV systolic function, as indicated by a lower absolute GLS magnitude (|GLS|). In the continuous models, the fixed-effect and random-effects estimates were comparable (I^2 ^= 0%), supporting the stability of this association. In categorical comparisons (Q4 vs. Q1), the direction of effect was consistent; however, heterogeneity was substantial, and therefore, random-effects estimates were prioritized. Prespecified subgroup analyses are presented in the Supplementary Materials and should be interpreted as exploratory, given the limited number of studies. These complementary approaches, which use both categorical (Q4 vs. Q1) and continuous IR modeling, support an inverse association between higher IR and |GLS|. Although Egger’s test did not indicate small-study effects, its power was limited by the small number of included studies (<10), and the results should be interpreted with caution.

### 4.2 Heterogeneity and Methodological Considerations

We observed substantial heterogeneity in quartile-based comparisons (Q4 vs. Q1) of |GLS| (MD), whereas continuous models showed minimal heterogeneity. This heterogeneity probably reflects between-study differences in the characteristics of participants and baseline cardiometabolic risk profiles, including cohorts with type 2 diabetes [16,18,20], obesity [17], and broader cardiovascular risk factor populations [21]. Additionally, the IR metrics and their categorization were not uniform: most studies evaluated TyG [16,17,18,20], whereas one used the TG/HDL ratio [21], and the thresholds defining “high IR” varied across studies (TyG cutoffs ranging from about 8.97 to 9.83 and TG/HDL >3.75). The definitions of outcomes also differed, with subclinical dysfunction defined using |GLS| cutoffs of <18% or <20%, which may contribute to variability in the magnitude of effects. Moreover, covariate adjustment sets varied across studies, including differences in whether adiposity measures, blood pressure, lipid profiles, renal function markers, and cardiac structural indices were included, which may influence the residual association attributed to IR. Accordingly, we prioritized random-effects estimates for quartile-based analyses and interpreted subgroup findings with caution, given the limited number of contributing studies.

### 4.3 Underlying Mechanisms

Several pathophysiological pathways may explain the observed association between higher IR and subclinical ventricular dysfunction. IR is accompanied by compensatory hyperinsulinemia, impaired uptake of glucose in cardiomyocytes, and a shift in substrate utilization toward increased oxidation of fatty acids, resulting in reduced metabolic flexibility and decreased cardiac efficiency [26]. This metabolic shift is energetically less efficient and promotes the accumulation of toxic lipid intermediates such as ceramides and diacylglycerols, which can trigger mitochondrial dysfunction, oxidative stress, endoplasmic reticulum stress, and apoptosis [27]. Moreover, IR is associated with the activation of the renin-angiotensin-aldosterone system and the sympathetic nervous system and with the upregulation of profibrotic signaling [26]. These processes favor diffuse interstitial fibrosis and concentric remodeling of the ventricle [26]. As subendocardial longitudinal fibers are particularly sensitive to metabolic and ischemic stress, longitudinal strain deteriorates before ejection fraction, which is consistent with our finding that IR is already associated with impaired |GLS| in populations with largely preserved EF [15,28]. Endothelial dysfunction and microvascular disease provide an additional link between IR and myocardial impairment. IR is associated with a decrease in nitric oxide bioavailability, an increase in vasoconstrictor tone, and structural rarefaction of the microcirculation [29]. These changes impair coronary flow reserve and may preferentially affect the subendocardial layers, thereby contributing to early abnormalities in longitudinal strain [30]. Systemic low-grade inflammation and high oxidative stress, both of which are common in IR states, may further damage cardiomyocytes and alter the extracellular matrix [6,31]. For the right ventricle, IR often coexists with central obesity, sleep-disordered breathing, and pulmonary vascular dysregulation, all of which can increase pulmonary pressure and impose additional loads on the right ventricle [32,33,34]. The evidence for right ventricular strain was limited and was not quantitatively synthesized; therefore, RV-specific mechanisms are speculative and require confirmation in future studies.

### 4.4 Fat Distribution and WHR

Fat distribution may further modulate myocardial vulnerability in insulin-resistant states [35,36,37]. The waist-to-hip ratio (WHR), a surrogate of visceral adiposity and android fat distribution, is associated with distinct cardiac phenotypes in previous studies, with android adiposity associated with greater subclinical myocardial dysfunction and gynoid fat distribution being relatively protective [38]. Although WHR was not consistently reported in the included studies and therefore could not be examined quantitatively in our meta-analysis, future observational and interventional studies incorporating WHR may help researchers determine whether the IR–|GLS| association is partly mediated by visceral adiposity, metabolic inflammation, and early myocardial remodeling [39]. Differences in visceral adiposity and fat distribution across cohorts may also contribute to between-study heterogeneity in the magnitude of |GLS| impairment [35].

### 4.5 Differences Between IR Indices and Between Clinical Subgroups

Differences between IR indices and between clinical subgroups are also biologically plausible. TyG indices combine fasting glucose and triglyceride levels in a single metric and therefore capture both IR and an atherogenic lipid environment, which may partly explain the slightly stronger association we observed for TyG than for some other indices [10]. Other composite scores, such as TG/HDL, capture related but not identical dimensions of metabolic dysfunction; although all are associated with visceral adiposity, ectopic fat accumulation, and impaired insulin signaling that ultimately converge on similar myocardial pathways, their metabolic components are weighted differently and may yield a slightly less pronounced gradient at the individual index level [40,41]. The slightly greater decrease in |GLS| observed in individuals with type 2 diabetes than in non-diabetic individuals is compatible with the idea that chronic hyperglycemia, the formation of advanced glycation end products, and diabetic microangiopathy act in addition to IR to amplify myocardial stiffness and fibrosis [42]. Moreover, the presence of a robust association in non-diabetic adults indicates that IR represents an adverse cardiac phenotype, rather than simply a bystander in the progression to overt diabetes. Given the limited number of studies within each subgroup and for each IR index, these differences between subgroups and indices should be considered hypothesis-generating rather than definitive.

### 4.6 Clinical Implications

Our findings indicate that IR is an early and clinically relevant cardiometabolic risk phenotype associated with subclinical LV systolic impairment before overt heart failure. Given that several IR indices (for example, TyG and TG/HDL) can be derived from routinely available laboratory measures, they may complement traditional risk assessment strategies by identifying individuals who might benefit from earlier lifestyle and cardiometabolic interventions. However, whether improving insulin sensitivity improves |GLS| or reduces clinical events remains to be established; future longitudinal and interventional studies should directly test this hypothesis.

### 4.7 Strengths and Limitations

This meta-analysis provides a quantitative synthesis of the available evidence on the associations between IR indices and subclinical left ventricular systolic dysfunction assessed by speckle-tracking echocardiography. By pooling results across seven studies and incorporating fully adjusted estimates, our analysis summarized the direction and magnitude of these associations, which individual studies are often underpowered to estimate precisely. These findings support further assessment of IR metrics as potential markers for early risk stratification, especially in settings where advanced diagnostic pathways are limited.

This study had several limitations. First, the included evidence was predominantly observational, which limited causal interpretation and did not eliminate residual confounding, even when multivariate adjustment was reported. Second, with only seven studies, the power to detect and fully characterize publication bias was constrained, and small-study effects may have influenced the pooled estimates. Third, methodological heterogeneity is likely, including differences in the definition and calculation of IR indices, patient characteristics, echocardiographic acquisition protocols, and strain-analysis software or vendor platforms, all of which may contribute to variability between studies. Finally, the limited number of studies restricted the scope and robustness of subgroup and sensitivity analyses; therefore, these analyses should be regarded as exploratory. Larger prospective cohorts using standardized strain protocols and harmonized IR definitions are needed to confirm these associations and to determine whether IR indices improve clinical prediction beyond established cardiometabolic risk factors. Additionally, right ventricular strain was reported in only one study; therefore, right ventricular findings were summarized narratively and should be considered preliminary.

## 5. Conclusions

Higher IR, as assessed by routinely available indices, is independently associated with worse subclinical LV systolic function in adults, reflected by lower absolute LV GLS magnitude (|GLS|). The association is directionally consistent across different IR metrics, although the evidence base remains limited, and subgroup findings should be interpreted cautiously. These results support IR as an early cardiac risk phenotype and highlight the need for prospective studies to determine whether interventions that improve insulin sensitivity can translate into improvements in myocardial strain and downstream clinical outcomes.

## Data Availability

All data generated or analyzed during this study are included in this published article and its **supplementary files**.
