# Supplementary material for: Insulin Resistance Indices and Subclinical Left Ventricular Systolic Dysfunction in Adults: A Systematic Review and Meta-Analysis
Source: Rev Cardiovasc Med. 2026 Jul 24;27(7):49364. doi: 10.31083/RCM49364 (PMC13419991; doi:10.31083/RCM49364)
Supplement: Supplementary file 1 [file 2153-8174-27-7-49364-s1.zip › Supplementary Material.docx]

**Additional file**

**Search strategies**

1. ***Embase***

('insulin resistance'/exp

OR 'insulin resistance':ti,ab

OR 'insulin resistant':ti,ab

OR 'HOMA-IR':ti,ab

OR 'homeostasis model assessment':ti,ab

OR 'homeostatic model assessment':ti,ab

OR 'triglyceride glucose index':ti,ab

OR 'triglyceride-glucose index':ti,ab

OR 'tyg index':ti,ab

OR 'METS-IR':ti,ab

OR 'metabolic score for insulin resistance':ti,ab

OR 'triglyceride to hdl cholesterol ratio':ti,ab

OR 'tg/hdl-c':ti,ab

OR 'tg hdl ratio':ti,ab

OR 'estimated glucose disposal rate':ti,ab)

AND

('ventricular dysfunction'/exp

OR 'ventricular function'/exp

OR 'global longitudinal strain':ti,ab

OR 'myocardial strain':ti,ab

OR 'longitudinal strain':ti,ab

OR 'left ventricular strain':ti,ab

OR 'right ventricular strain':ti,ab

OR 'right ventricular free wall':ti,ab

OR 'rv free wall':ti,ab)

AND

('echocardiography'/exp

OR echocardiograph*:ti,ab

OR 'speckle tracking':ti,ab

OR 'speckle-tracking':ti,ab

OR 'speckle tracking echocardiography':ti,ab

OR 'strain echocardiography':ti,ab

OR 'strain imaging':ti,ab)

AND [humans]/lim

AND [adult]/lim

AND [article]/lim

AND [1966-2025]/py

1. ***Web of Science***

TS = (("insulin resistance"

OR "insulin-resistant"

OR "HOMA-IR"

OR "homeostasis model assessment"

OR "homeostatic model assessment"

OR "triglyceride-glucose index"

OR "triglyceride glucose index"

OR "TyG index"

OR "METS-IR"

OR "metabolic score for insulin resistance"

OR "triglyceride to HDL cholesterol ratio"

OR "TG/HDL-C"

OR "TG HDL ratio"

OR "estimated glucose disposal rate")

AND

("global longitudinal strain"

OR "myocardial strain"

OR "longitudinal strain"

OR "left ventricular strain"

OR "right ventricular strain"

OR "right ventricular free wall"

OR "RV free wall")

AND

("echocardiography"

OR "speckle tracking"

OR "speckle-tracking"

OR "speckle tracking echocardiography"

OR "strain echocardiography"

OR "strain imaging"))

1. ***Pubmed:***

(("Insulin Resistance"[Mesh]

OR "Insulin Resistance"[tiab]

OR insulin-resistant[tiab]

OR "HOMA-IR"[tiab]

OR "homeostasis model assessment"[tiab]

OR "homeostatic model assessment"[tiab]

OR "triglyceride-glucose index"[tiab]

OR "triglyceride glucose index"[tiab]

OR "TyG index"[tiab]

OR "METS-IR"[tiab]

OR "metabolic score for insulin resistance"[tiab]

OR "triglyceride to HDL cholesterol ratio"[tiab]

OR "TG/HDL-C"[tiab]

OR "TG HDL ratio"[tiab]

OR "estimated glucose disposal rate"[tiab])

AND

("Ventricular Dysfunction, Left"[Mesh]

OR "Ventricular Dysfunction, Right"[Mesh]

OR "Ventricular Function, Left"[Mesh]

OR "Ventricular Function, Right"[Mesh]

OR "global longitudinal strain"[tiab]

OR "myocardial strain"[tiab]

OR "longitudinal strain"[tiab]

OR "left ventricular strain"[tiab]

OR "right ventricular strain"[tiab]

OR "right ventricular free wall"[tiab]

OR "RV free wall"[tiab])

AND

("Echocardiography"[Mesh]

OR echocardiography[tiab]

OR "speckle tracking"[tiab]

OR "speckle-tracking"[tiab]

OR "speckle tracking echocardiography"[tiab]

OR "strain echocardiography"[tiab]

OR "strain imaging"[tiab]))

AND Humans[Mesh]

AND "adult"[MeSH Terms]

AND ("1900/01/01"[Date - Publication] : "2025/12/01"[Date - Publication])

***4, Cochrane Library***

#1 MeSH descriptor: [Insulin Resistance] explode all trees

#2 (insulin resistance OR HOMA-IR OR "homeostasis model assessment"

OR "triglyceride-glucose index" OR "triglyceride glucose index"

OR "TyG index" OR "METS-IR" OR "metabolic score for insulin resistance"

OR "triglyceride to HDL cholesterol ratio" OR "TG/HDL-C"

OR "TG HDL ratio" OR "estimated glucose disposal rate"):ti,ab,kw

#3 #1 OR #2

#4 MeSH descriptor: [Ventricular Dysfunction, Left] explode all trees

#5 MeSH descriptor: [Ventricular Dysfunction, Right] explode all trees

#6 (global longitudinal strain OR myocardial strain OR "left ventricular strain"

OR "right ventricular strain" OR "right ventricular free wall"

OR "RV free wall"):ti,ab,kw

#7 MeSH descriptor: [Echocardiography] explode all trees

#8 (speckle tracking OR speckle-tracking

OR "speckle tracking echocardiography"

OR "strain echocardiography" OR "strain imaging"):ti,ab,kw

#9 #4 OR #5 OR #6

#10 #7 OR #8

#11 #3 AND #9 AND #10

Supplementary Table 1 MODIFIED NEWCASTLE - OTTAWA QUALITY ASSESSMENT SCALE FOR CROSS - SECTIONAL

| **Item** | **English criteria** |
| --- | --- |
| **1. Representativeness of the sample** | **1 star**: The sample is truly or somewhat representative of the target population. **0 star**: The sample is a clearly selected group, or representativeness is not described. |
| **2. Sample size justification** | **1 star**: Sample size is large and/or a formal sample size calculation or power justification is reported. **0 star**: No sample size justification and the sample is clearly small or not discussed. |
| **3. Handling of non-respondents / exclusions** | **1 star**: Response rate is reported and reasonably high, or characteristics of responders and non-responders / excluded participants are compared and shown to be similar. **0 star**: Response rate is low, not reported, or there is no information comparing responders with non-responders / excluded participants. |
| **4. Ascertainment of exposure** | **1 star**: The exposure of interest is measured using standardized, validated methods, based on objective laboratory tests, with clear definitions. **0 star**: Exposure is based only on self-report without validation, or methods are poorly described. |
| **5. Comparability of subjects on the basis of design or analysis** | **Up to 2 stars**: - **1 star** if the study controls for the most important confounder(s). - **+1 star** if the study further controls for additional key cardiovascular or metabolic risk factors through multivariable analysis or matching. - **0 star** if there is no adjustment for confounders, or only crude comparisons are reported. |
| **6. Assessment of outcome** | **1 star**: Outcome is assessed using standardized echocardiographic protocols and validated speckle-tracking software, with clear definitions and blinded or independent assessment when possible. **0 star**: Outcome assessment is not clearly described, not standardized, or based on non-validated or subjective measures. |
| **7. Outcome definition and cut-off** | **1 star**: The study provides a clear and clinically or guideline-based definition of subclinical ventricular dysfunction and applies it consistently. **0 star**: The definition of outcome is unclear, arbitrary, or not referenced, or thresholds are inconsistently applied. |
| **8. Statistical analysis and reporting** | **1 star**: Appropriate statistical tests are used, the association between exposure and outcome is quantified, model assumptions are reasonably addressed, and precision estimates are reported. **0 star**: Statistical methods are inappropriate, not described in sufficient detail, or key estimates of association and variability are missing. |

Supplementary Table 2 Modified NOS of seven studies

| **Author** | **Selection**  **(0–4)** | **Comparability**  **(0–2)** | **Outcome**  **(0–3)** | **Total**  **(0–9)** |
| --- | --- | --- | --- | --- |
| SUN et al. | **3** | **2** | **3** | **8** |
| Li et al. | **4** | **2** | **3** | **9** |
| Chen et al. | **3** | **2** | **3** | **8** |
| Yang et al. | **3** | **2** | **3** | **8** |
| Zhou et al. | **3** | **2** | **3** | **8** |
| Cassano et al. | **3** | **1** | **3** | **7** |
| Bian et al. | **2** | **2** | **3** | **7** |


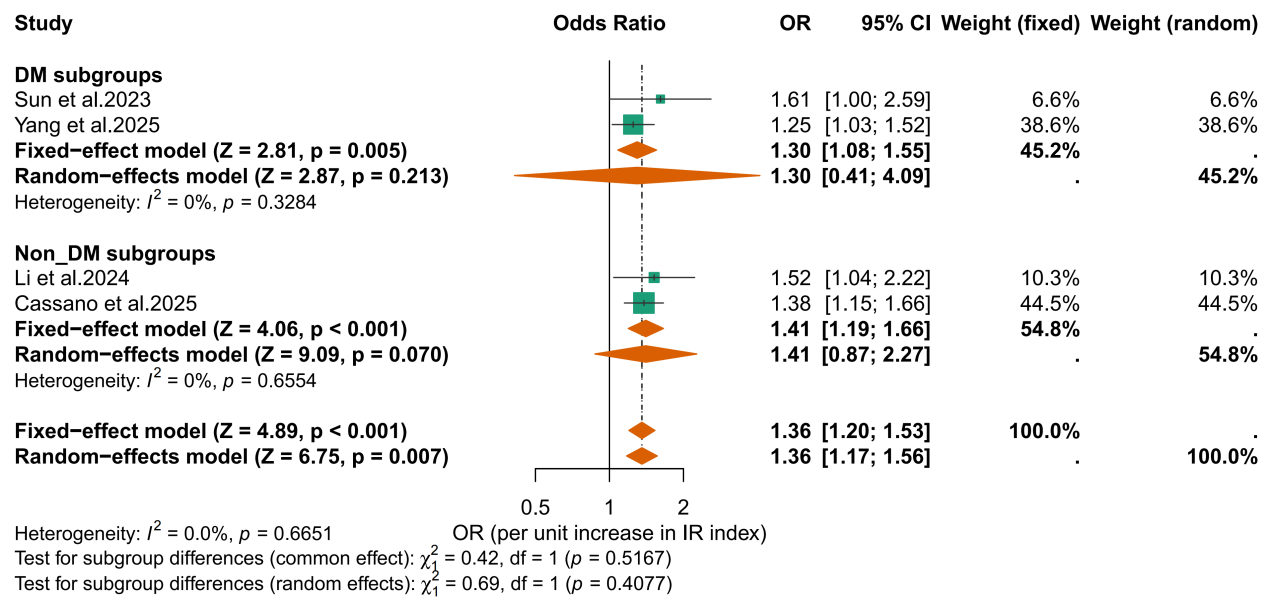


Supplementary Fig. 1 Subgroup forest plot of the association between IR indices and |GLS| in DM vs non-DM

**
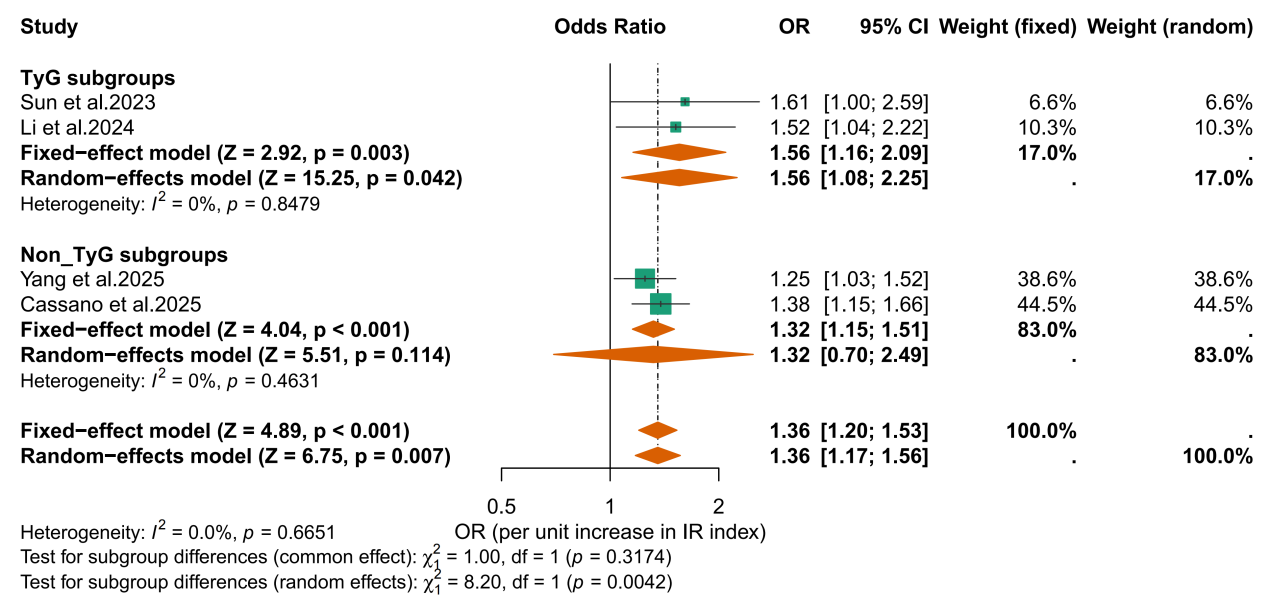
**

Supplementary Fig. 2 Subgroup forest plot of the association between IR indices and |GLS| in TyG and Non-TyG subgroup

**
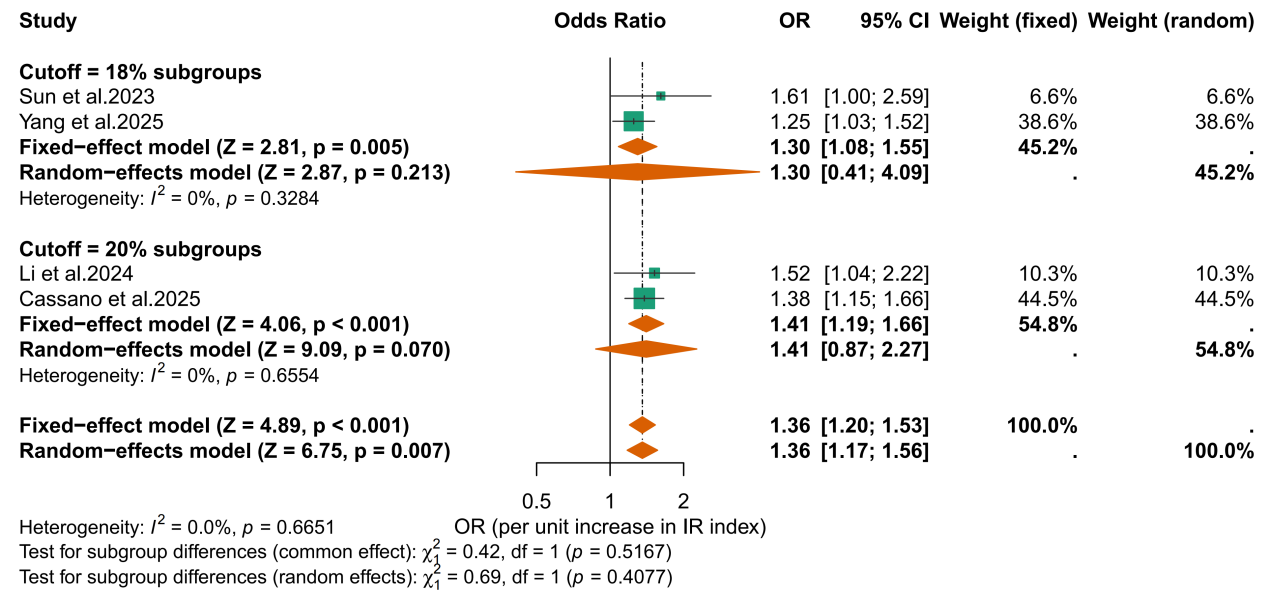
**

Supplementary Fig. 3 Subgroup forest plot of the association between IR indices and |GLS| in cutoff = 18% and cutoff = 20% subgroup
